# Supplementary material for: Comparative analysis of zebrafish fear responses to eight different fish species using three-dimensional locomotion-tracking assays
Source: Biol Open. 2025 Oct 8;14(10):bio062110. doi: 10.1242/bio.062110 (PMC12547860; doi:10.1242/bio.062110)
Supplement: Supplementary information [file biolopen-14-062110-s1.pdf]

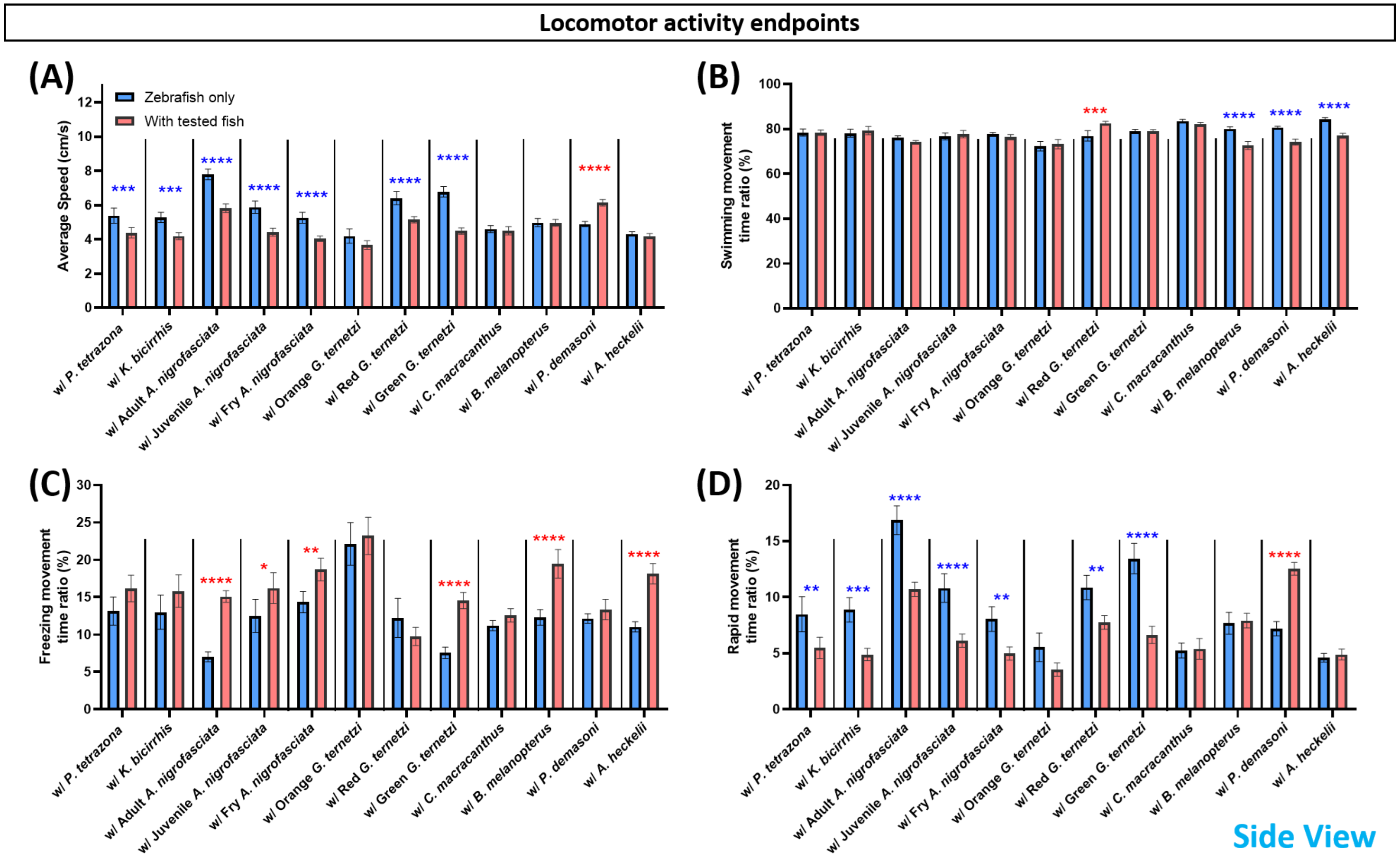


**Fig. S1. Comparison of zebrafish locomotor activity endpoints side view before (blue bar) and after introduction to tested fishes (red bar). Four endpoints were calculated in this group, (A) Average speed, (B) Swimming movement time ratio, (C) Freezing movement time ratio, and (D) Rapid movement time ratio.** Data were presented in a bar plot (Mean ± SEM) and processed using Two-way ANOVA mixed-effects analysis with uncorrected Fisher's LSD post hoc test (n = 4, with 6 zebrafish per replication for zebrafish only and with tested fish group; * $p < 0.05$, ** $p < 0.01$, *** $p < 0.001$, **** $p < 0.0001$. Red asterisk represents increased activity when tested fish was added to the tank in comparison to zebrafish only, while blue asterisk represents decreased activity).

Biology Open • Supplementary information

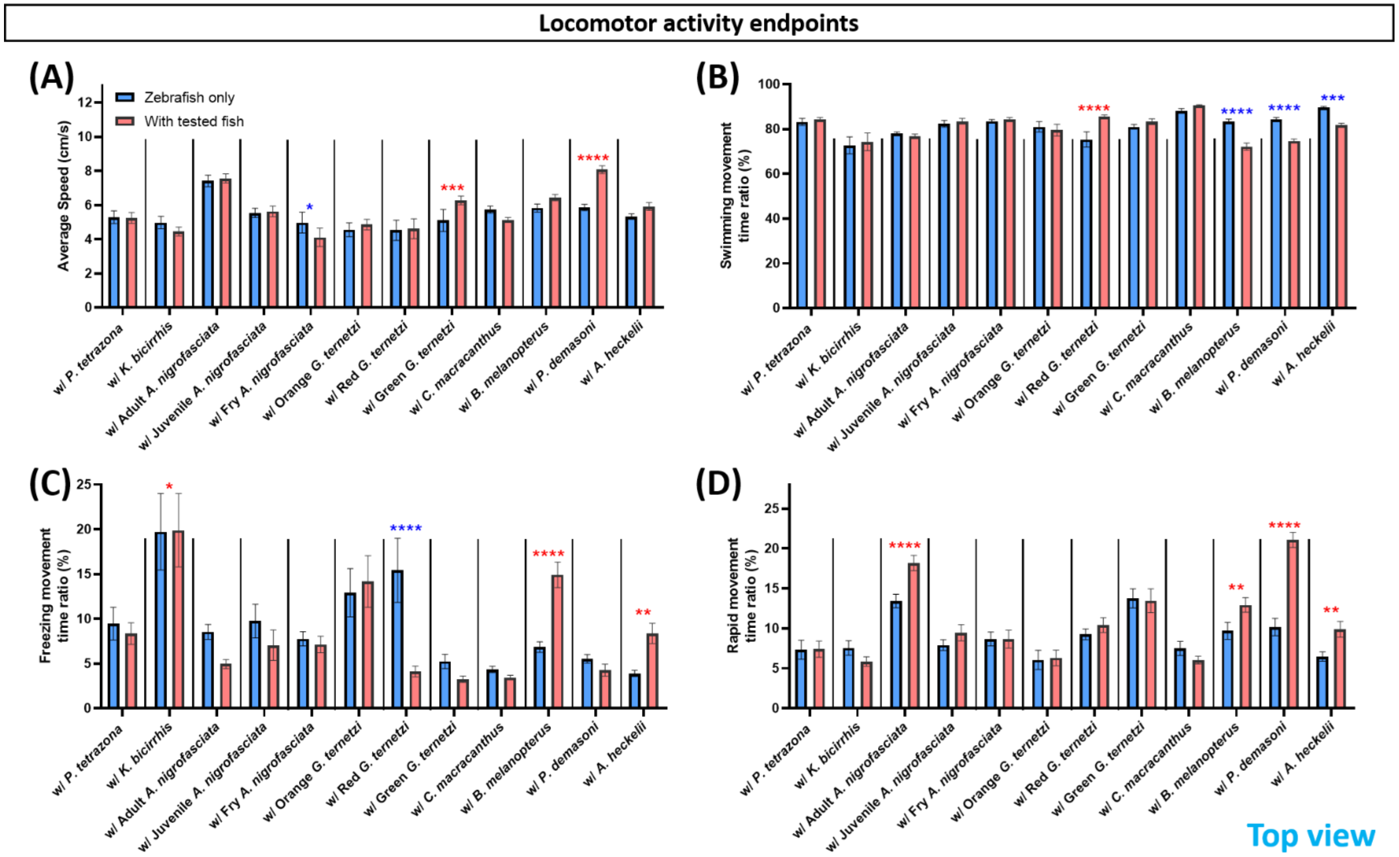


**Fig. S2. Comparison of zebrafish locomotor activity endpoints top view before (blue bar) and after introduction to tested fishes (red bar). Four endpoints were calculated in this group, (A) Average speed, (B) Swimming movement time ratio, (C) Freezing movement time ratio, and (D) Rapid movement time ratio.** Data were presented in a bar plot (Mean ± SEM) and processed using Two-way ANOVA mixed-effects analysis with uncorrected Fisher's LSD post hoc test (n = 4, with 6 zebrafish per replication for zebrafish only and with tested fish group; * $p < 0.05$, ** $p < 0.01$, *** $p < 0.001$, **** $p < 0.0001$. Red asterisk represents increased activity when tested fish was added to the tank in comparison to zebrafish only, while blue asterisk represents decreased activity).

Biology Open • Supplementary information

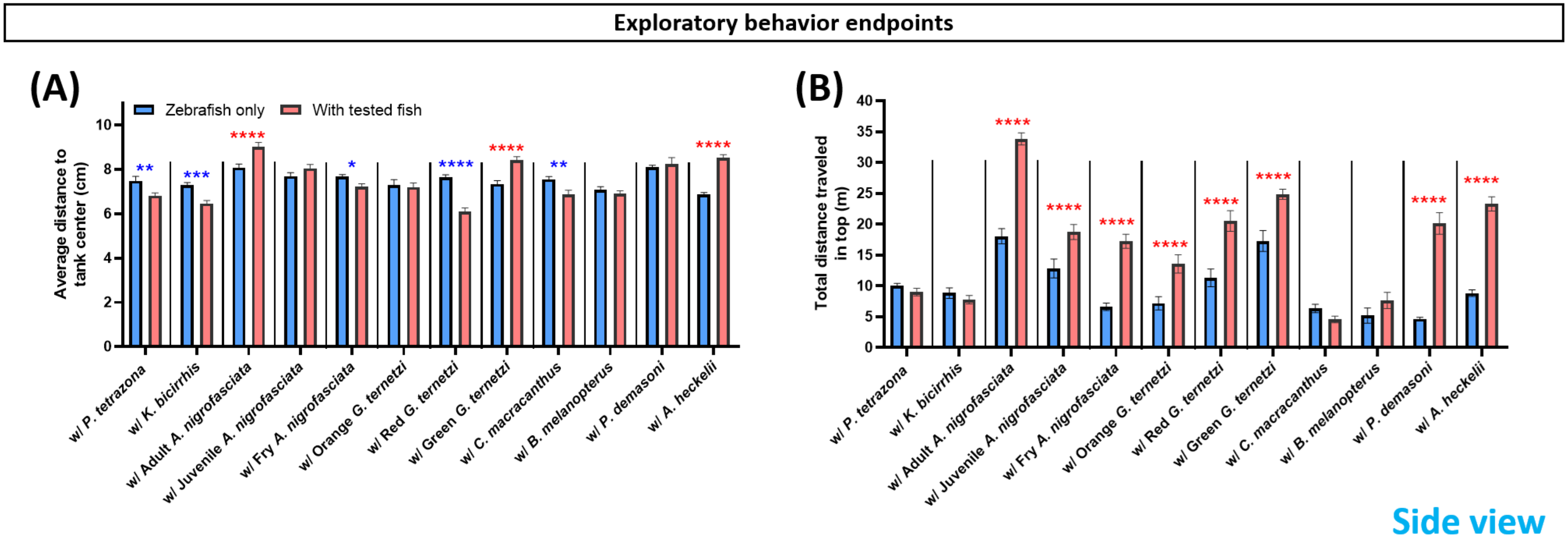


**Fig. S3. Comparison of zebrafish exploratory behavior endpoints in side view before (blue bar) and after introduction to tested fishes (red bar). Two endpoints were calculated in this group, (A) Average distance to tank center and (B) Total distance traveled in top.** Data were presented in a bar plot (Mean ± SEM) and processed using Two-way ANOVA mixed-effects analysis with uncorrected Fisher's LSD post hoc test (n = 4, with 6 zebrafish per replication for zebrafish only and with tested fish group; * $p < 0.05$, ** $p < 0.01$, *** $p < 0.001$, **** $p < 0.0001$. Red asterisk represents increased activity when tested fish was added to the tank in comparison to zebrafish only, while blue asterisk represents decreased activity).

Biology Open • Supplementary information

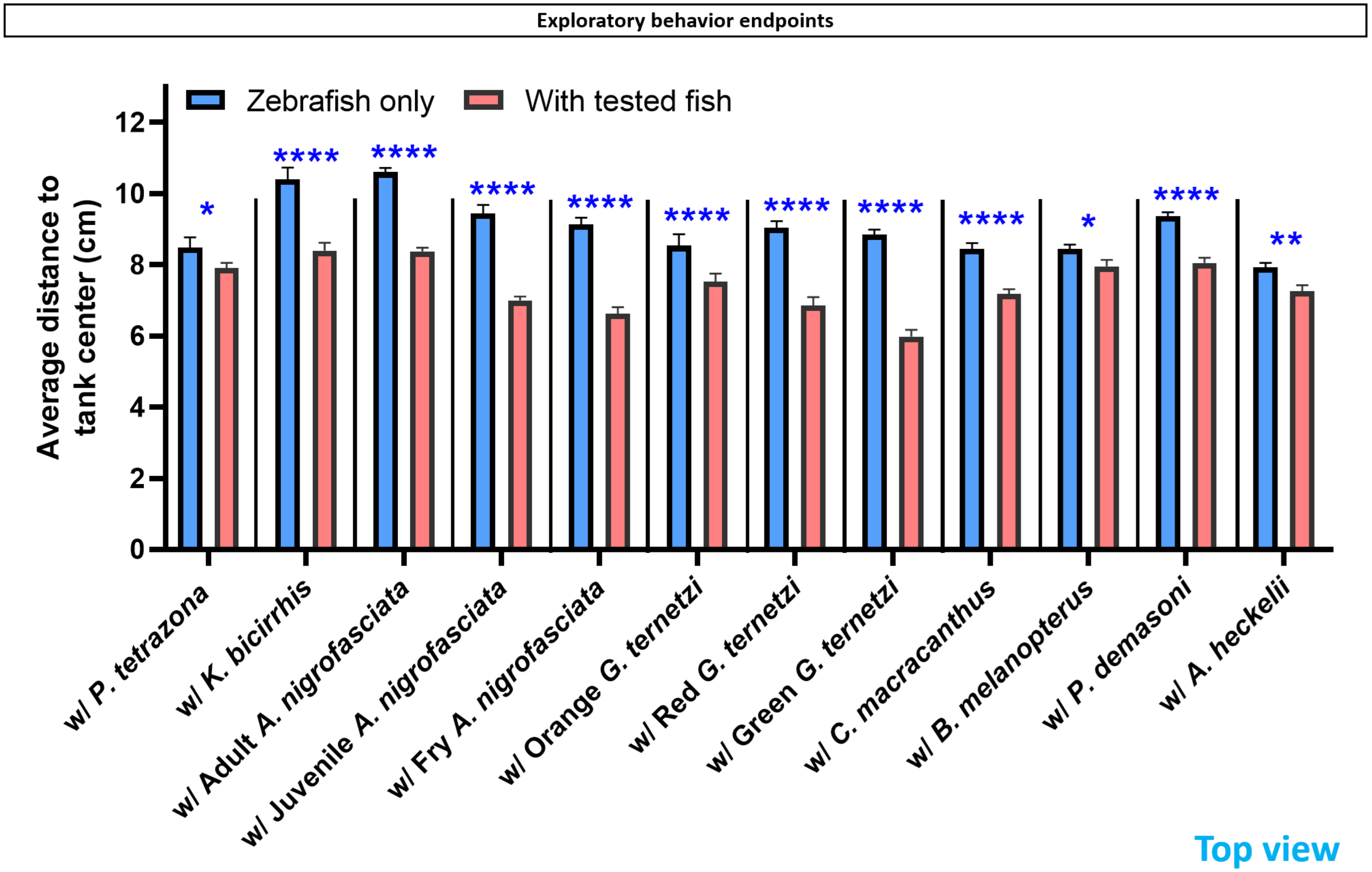


**Fig. S4. Comparison of zebrafish exploratory behavior endpoints in side view before (blue bar) and after introduction to tested fishes (red bar).** One endpoint was calculated in this group, Average distance to tank center. Data were presented in a bar plot (Mean ± SEM) and processed using Two-way ANOVA mixed-effects analysis with uncorrected Fisher's LSD post hoc test (n = 4, with 6 zebrafish per replication for zebrafish only and with tested fish group; * $p < 0.05$, ** $p < 0.01$, *** $p < 0.001$, **** $p < 0.0001$. Red asterisk represents increased activity when tested fish was added to the tank in comparison to zebrafish only, while blue asterisk represents decreased activity).

Biology Open • Supplementary information

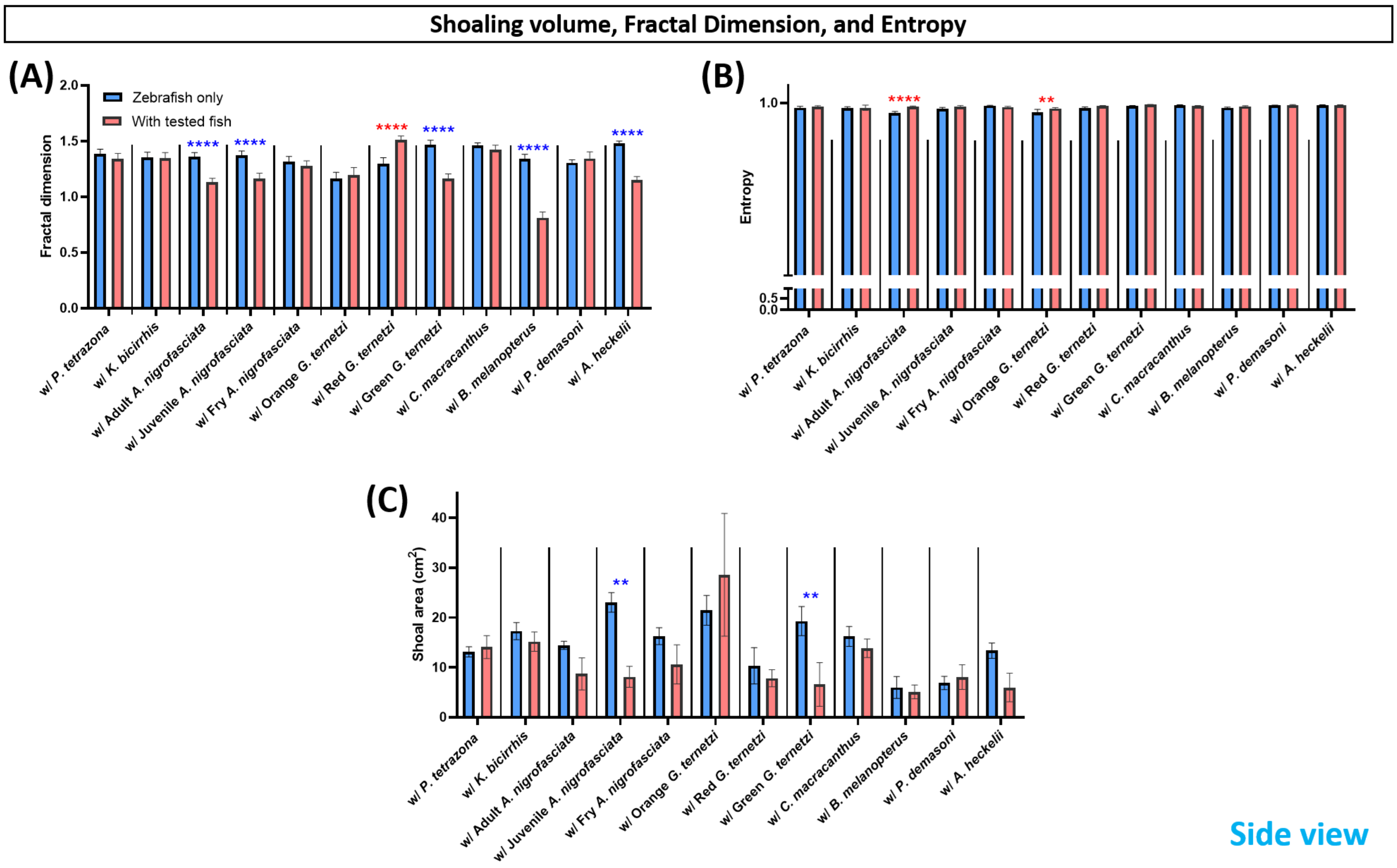


**Fig. S5. Comparison of zebrafish behavioral endpoints in side view before (blue bar) and after introduction to tested fishes (red bar). Three endpoints were calculated in this group, (A) Fractal dimension, (B) Entropy, and (C) Shoaling volume.** Data were presented in a bar plot (Mean ± SEM) and processed using Two-way ANOVA mixed-effects analysis with uncorrected Fisher's LSD post hoc test (n = 4, with 6 zebrafish per replication for fractal dimension and entropy endpoints, n = 4 for shoaling; * $p < 0.05$, ** $p < 0.01$, *** $p < 0.001$, **** $p < 0.0001$. Red asterisk represents increased activity when tested fish was added to the tank in comparison to zebrafish only, while blue asterisk represents decreased activity).

Biology Open • Supplementary information

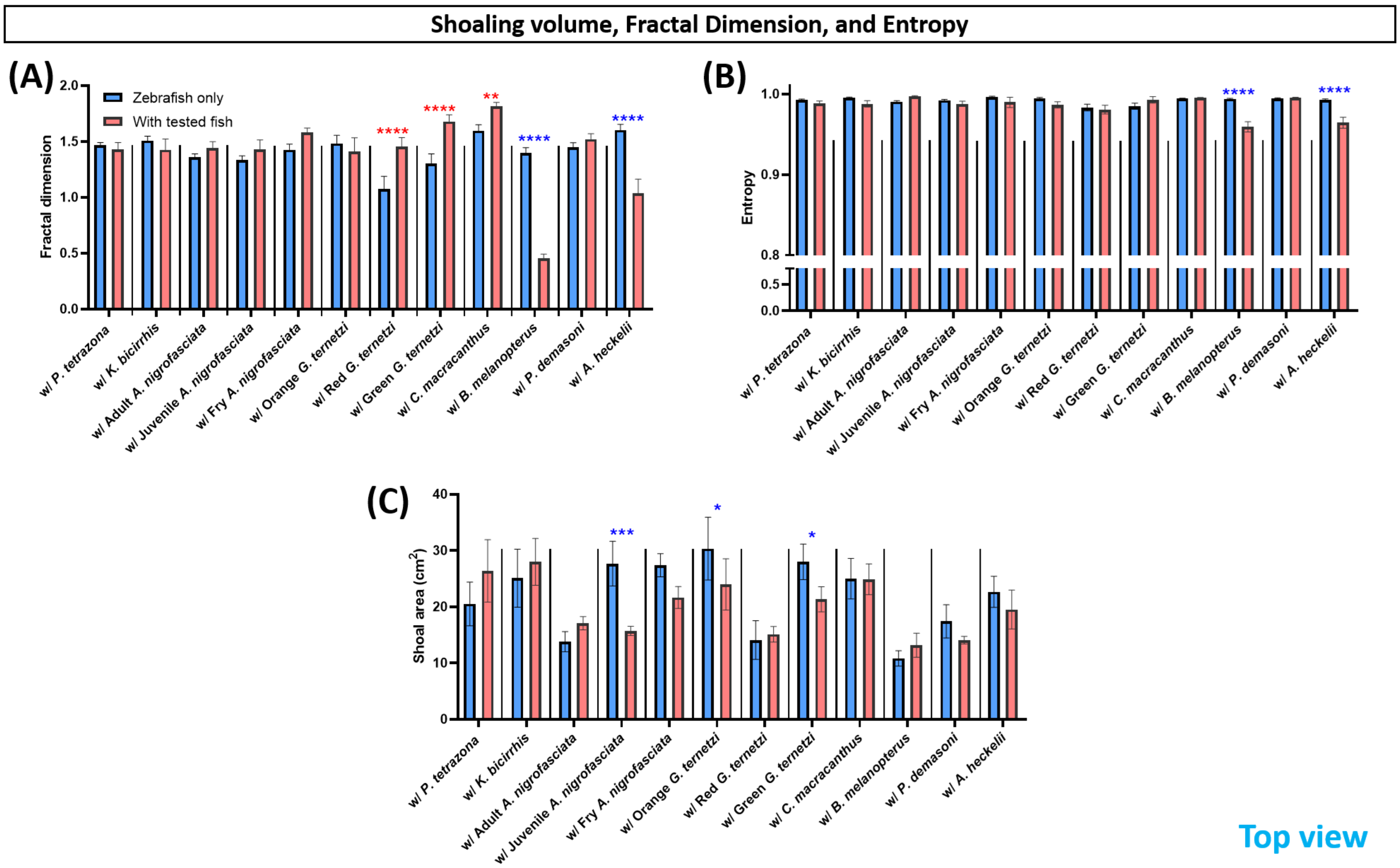


**Fig. S6. Comparison of zebrafish behavioral endpoints in top view before (blue bar) and after introduction to tested fishes (red bar). Three endpoints were calculated in this group, (A) Fractal dimension, (B) Entropy, and (C) Shoaling volume.** Data were presented in a bar plot (Mean ± SEM) and processed using Two-way ANOVA mixed-effects analysis with uncorrected Fisher's LSD post hoc test (n = 4, with 6 zebrafish per replication for fractal dimension and entropy endpoints, n = 4 for shoaling; * $p < 0.05$, ** $p < 0.01$, *** $p < 0.001$, **** $p < 0.0001$. Red asterisk represents increased activity when tested fish was added to the tank in comparison to zebrafish only, while blue asterisk represents decreased activity).

Biology Open • Supplementary information

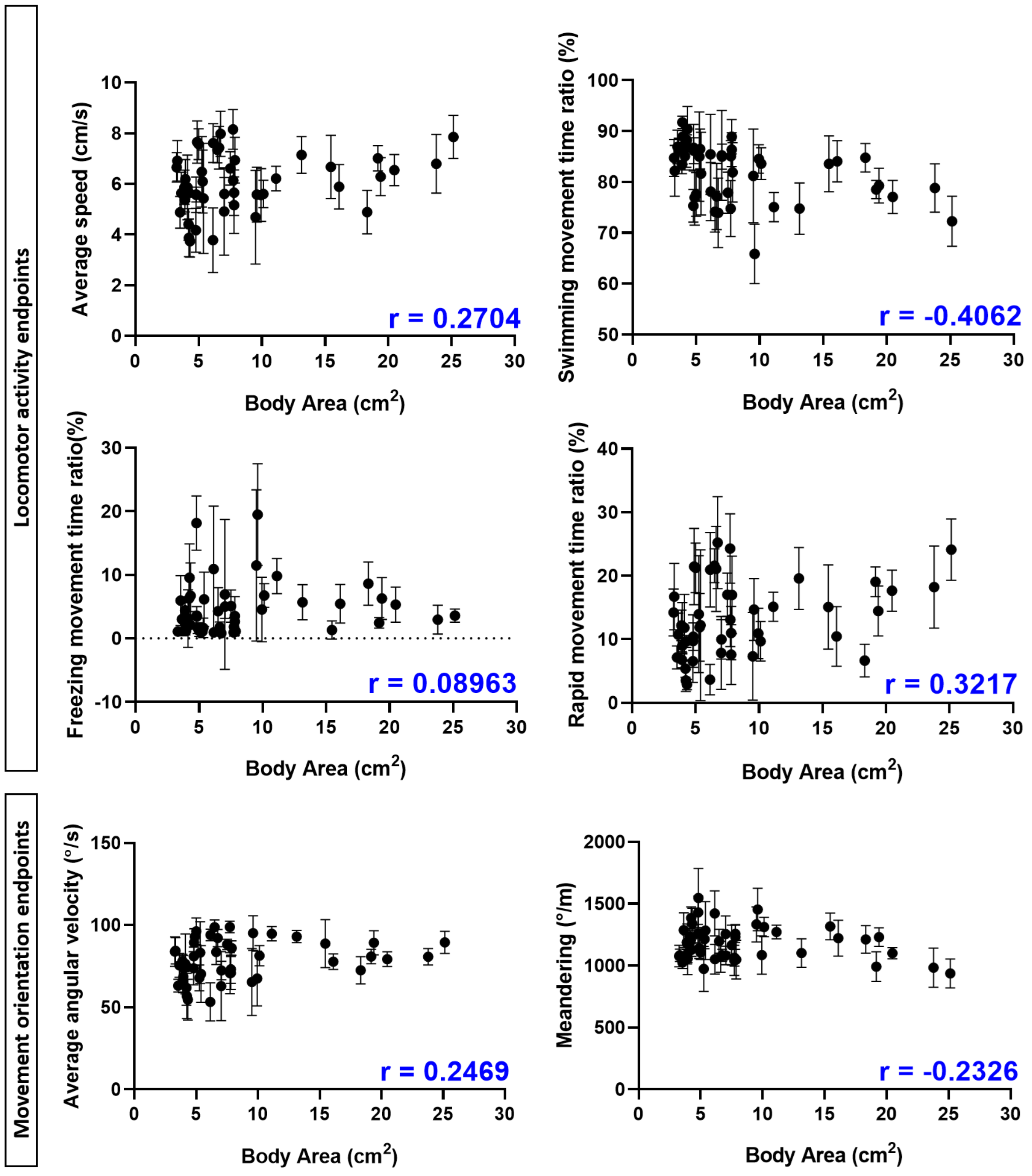


Biology Open • Supplementary information

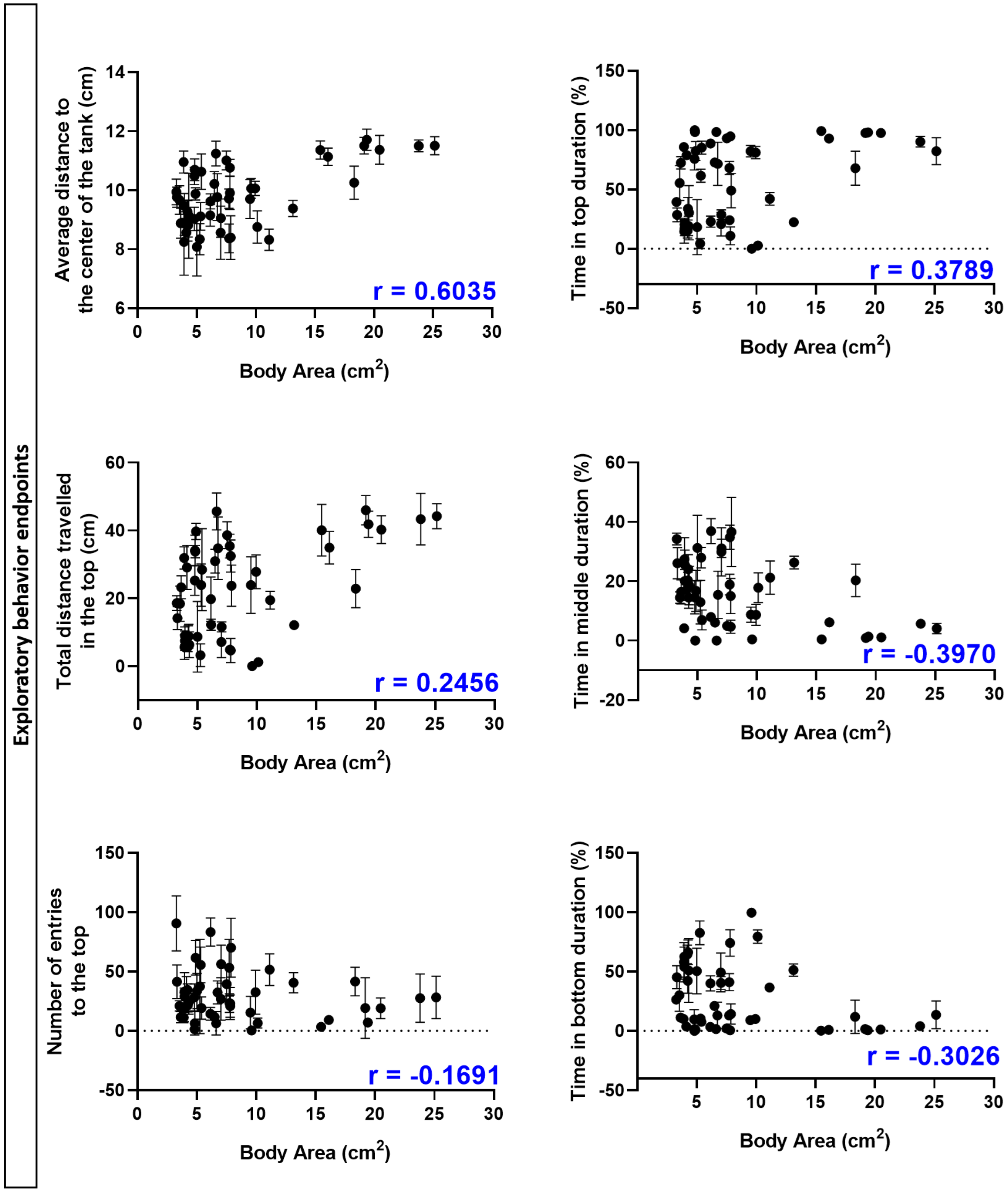


Biology Open • Supplementary information

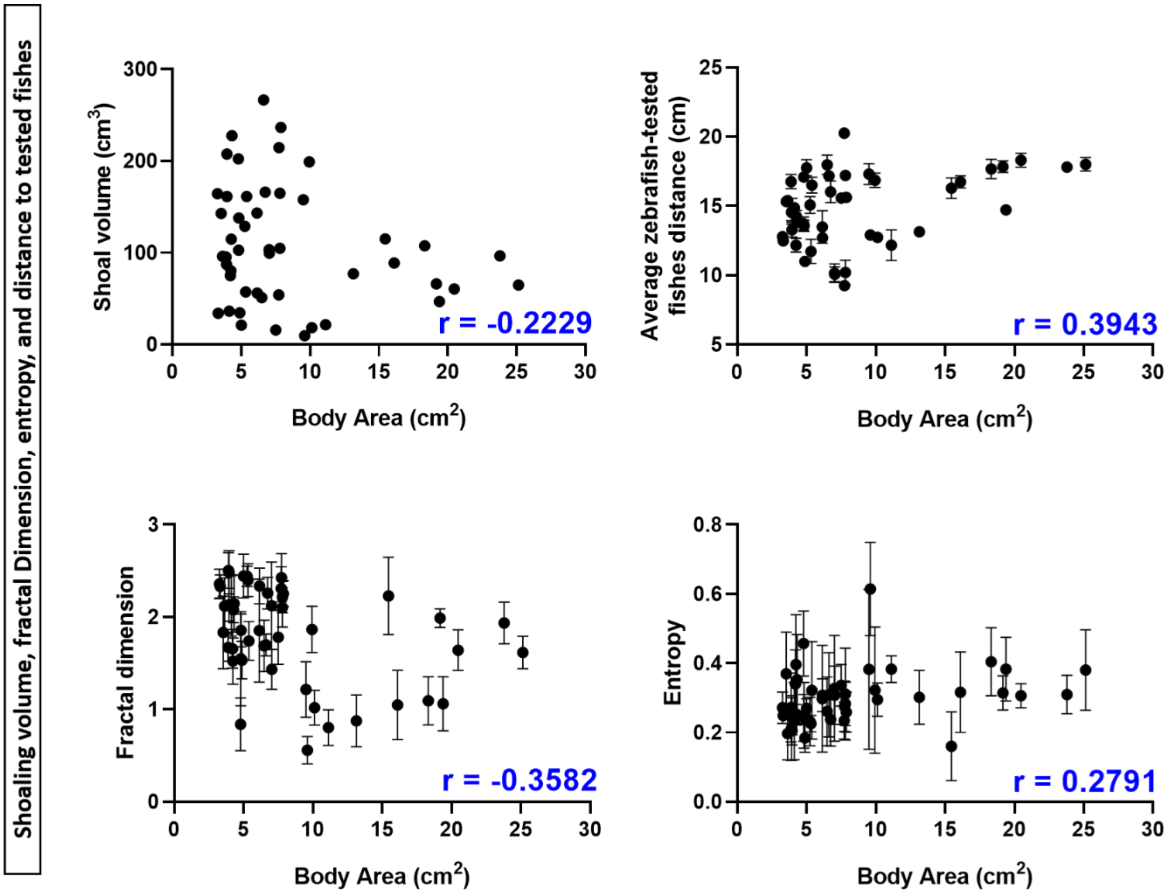


**Fig. S7. Pearson correlation (r) between tested fishes body area to all tested three-dimensional zebrafish behavior endpoints collected in shared environment test, data is presented as Mean ± SD (n = 48, 4 individuals per variation per species).**

Biology Open • Supplementary information

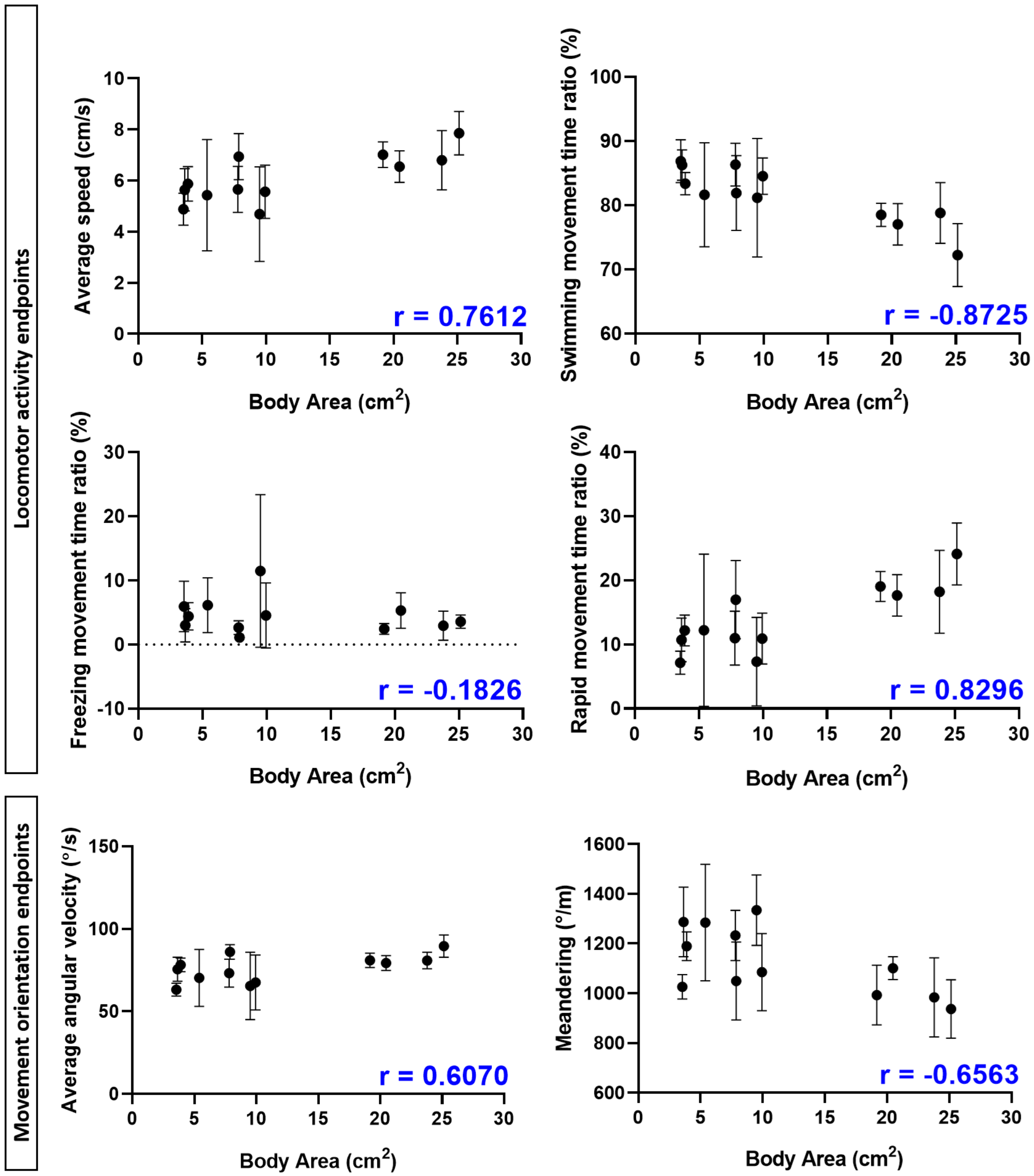
Locomotor activity endpoints
Average speed (cm/s)
r = 0.7612
Body Area (cm²)
Swimming movement time ratio (%)
r = -0.8725
Body Area (cm²)
Freezing movement time ratio (%)
r = -0.1826
Body Area (cm²)
Rapid movement time ratio (%)
r = 0.8296
Body Area (cm²)
Movement orientation endpoints
Average angular velocity (°/s)
r = 0.6070
Body Area (cm²)
Meandering (°/m)
r = -0.6563
Body Area (cm²)

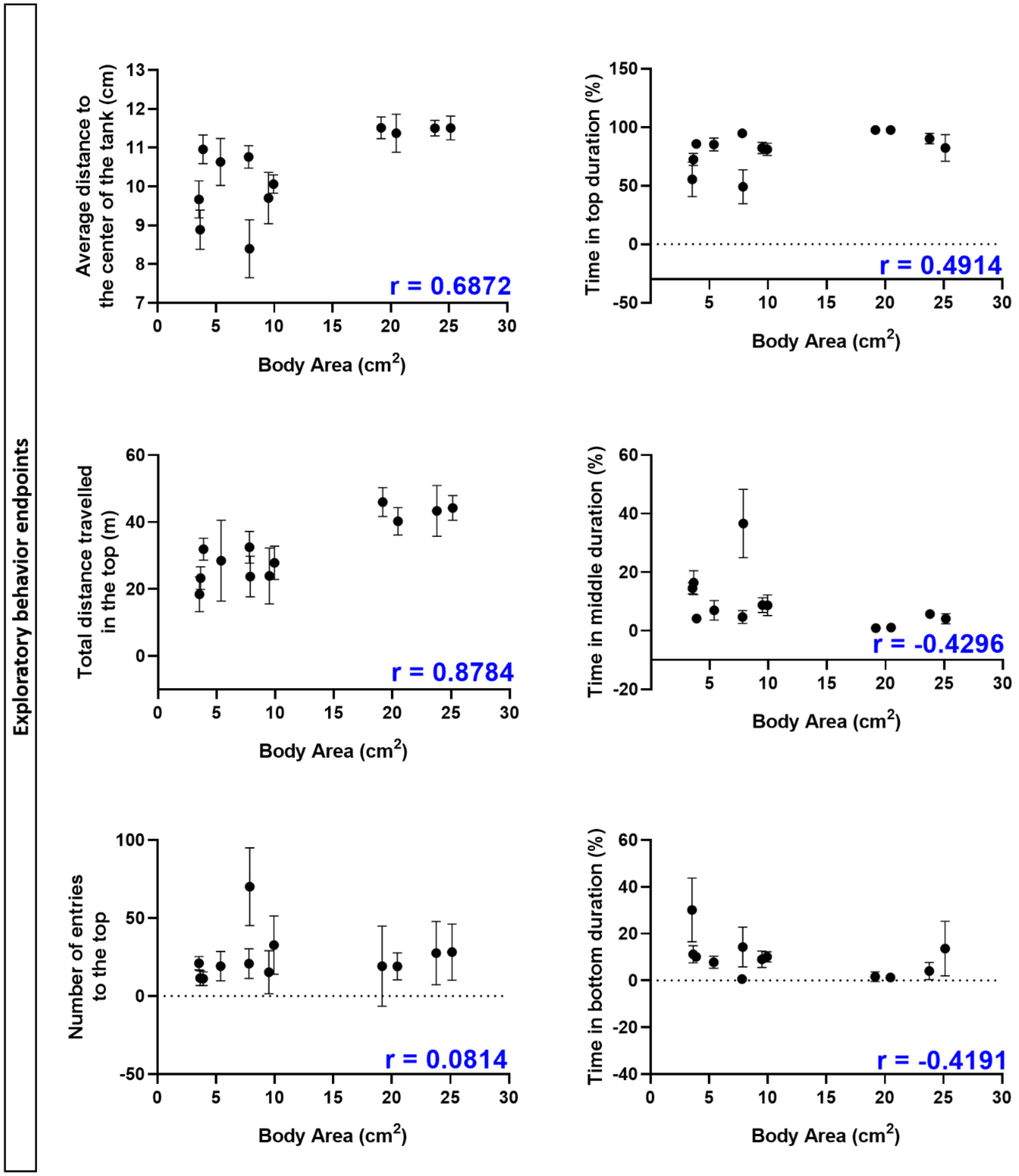
Exploratory behavior endpoints
Average distance to the center of the tank (cm)
r = 0.6872
Body Area (cm2)
Time in top duration (%)
r = 0.4914
Body Area (cm2)
Total distance travelled in the top (m)
r = 0.8784
Body Area (cm2)
Time in middle duration (%)
r = -0.4296
Body Area (cm2)
Number of entries to the top
r = 0.0814
Body Area (cm2)
Time in bottom duration (%)
r = -0.4191
Body Area (cm2)

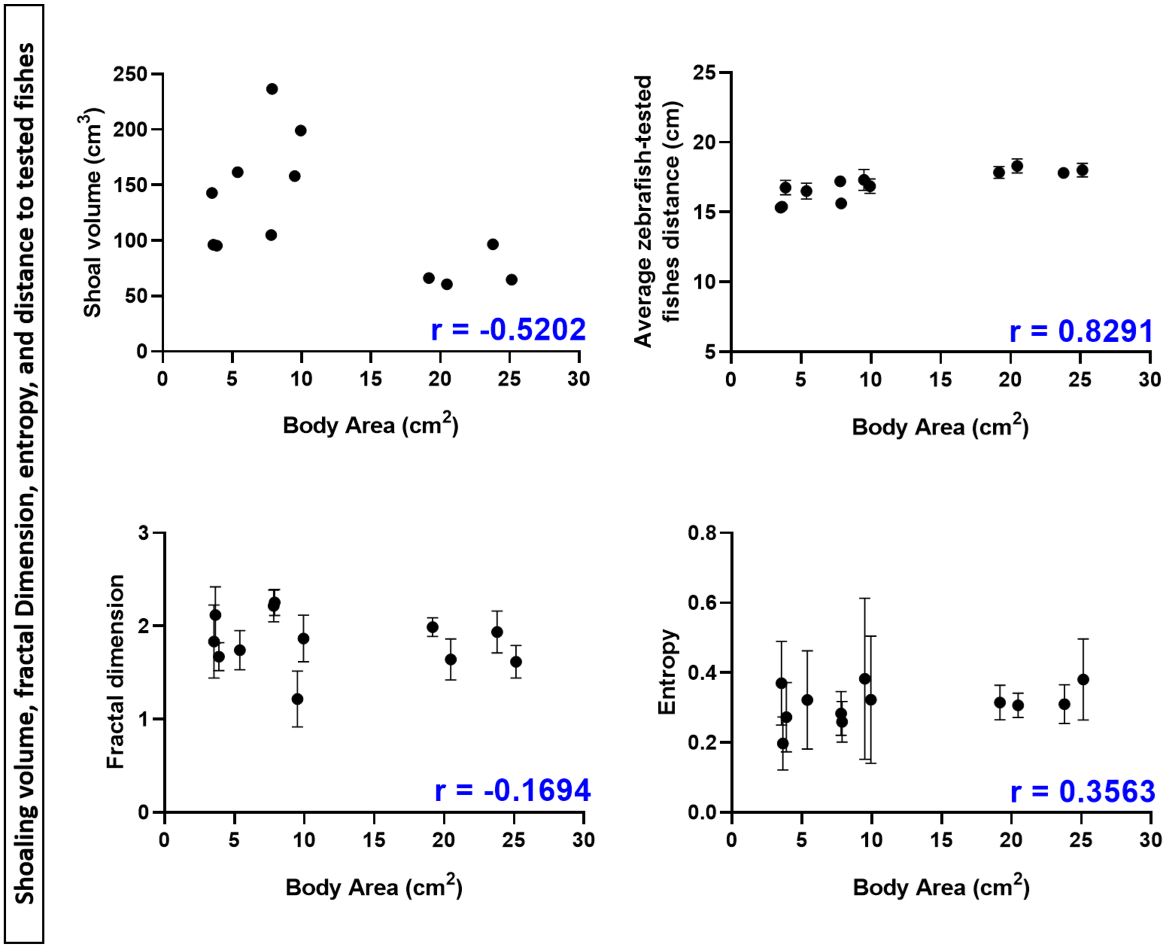


**Fig. S8. Pearson correlation (r) between convict cichlid body area at different growth stages to all tested three-dimensional zebrafish behavior endpoints in shared environment test, data is presented as Mean ± SD (n = 12, 4 individual per growth stage).**

Biology Open • Supplementary information

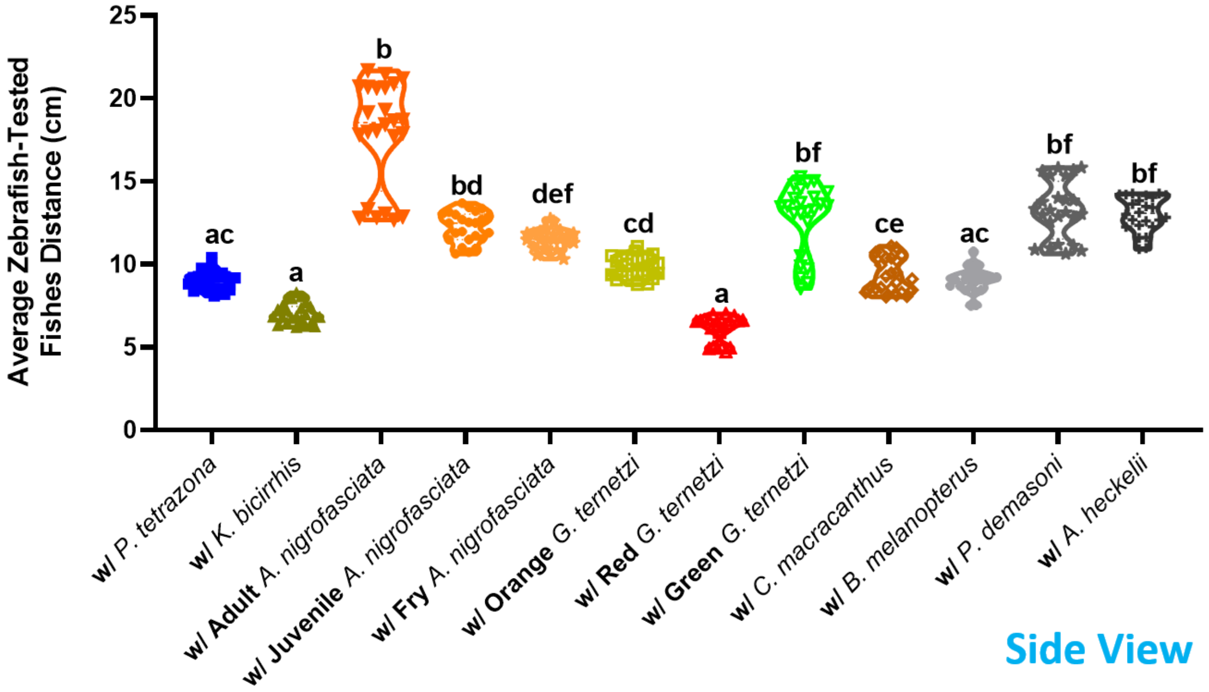


**Fig. S9. Comparison of average zebrafish to tested fishes distance in side view during 3D locomotion test.** Data were presented in a violin plot showing each point and processed using Kruskal-Wallis with Dunn's multiple comparison test (n = 4, with 6 zebrafish per replication; different letter represents a statistically significant difference, $p < 0.05$).

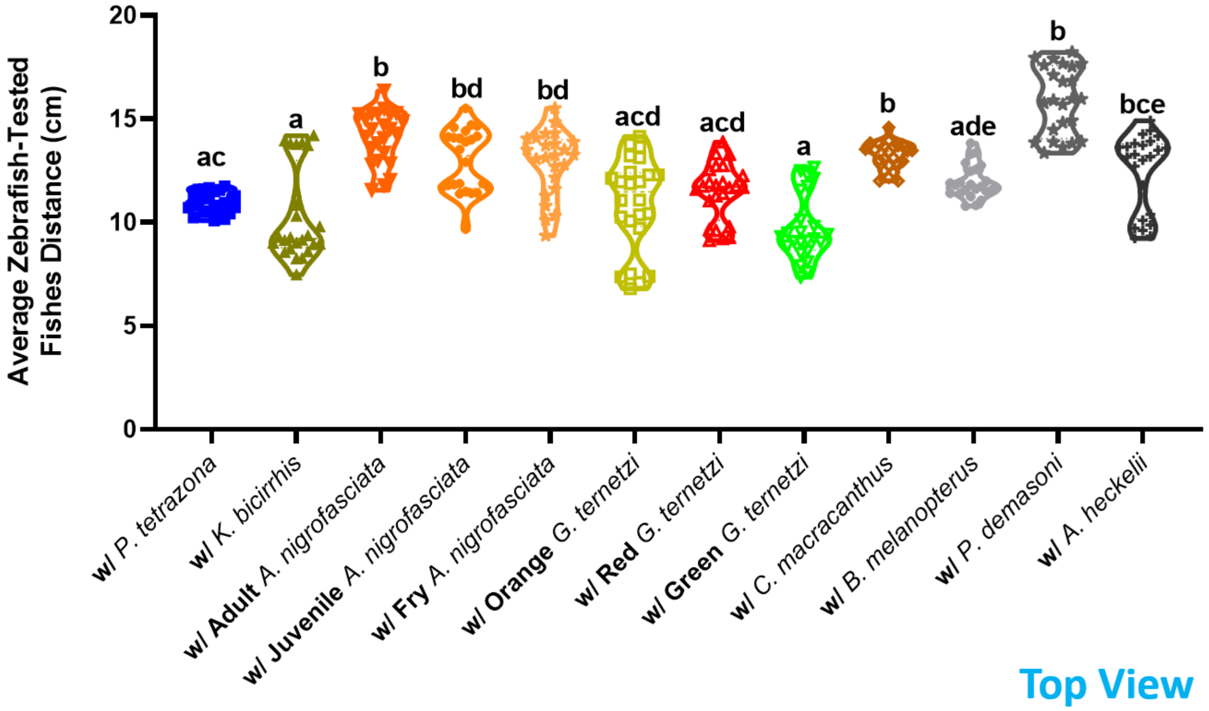


**Fig. S10. Comparison of Average zebrafish to tested fishes distance in top view during 3D locomotion test.** Data were presented in a violin plot showing each point and processed using Kruskal-Wallis with Dunn's multiple comparison test (n = 4, with 6 zebrafish per replication; different letter represents a statistically significant difference, $p < 0.05$).

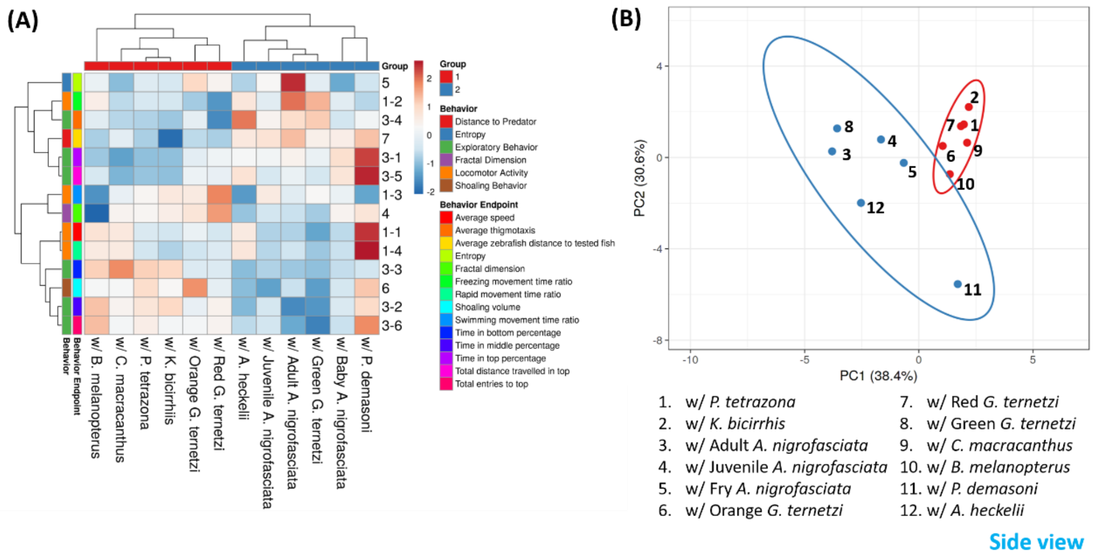


**Fig. S11. (A) Heatmap clustering analysis and (B) Principal component analysis (PCA) of all behavioral endpoints in zebrafish side view data after introduction to tested fishes.** Two major clusters were formed from the heatmap clustering marked with red (Group 1) and blue (Group 2).

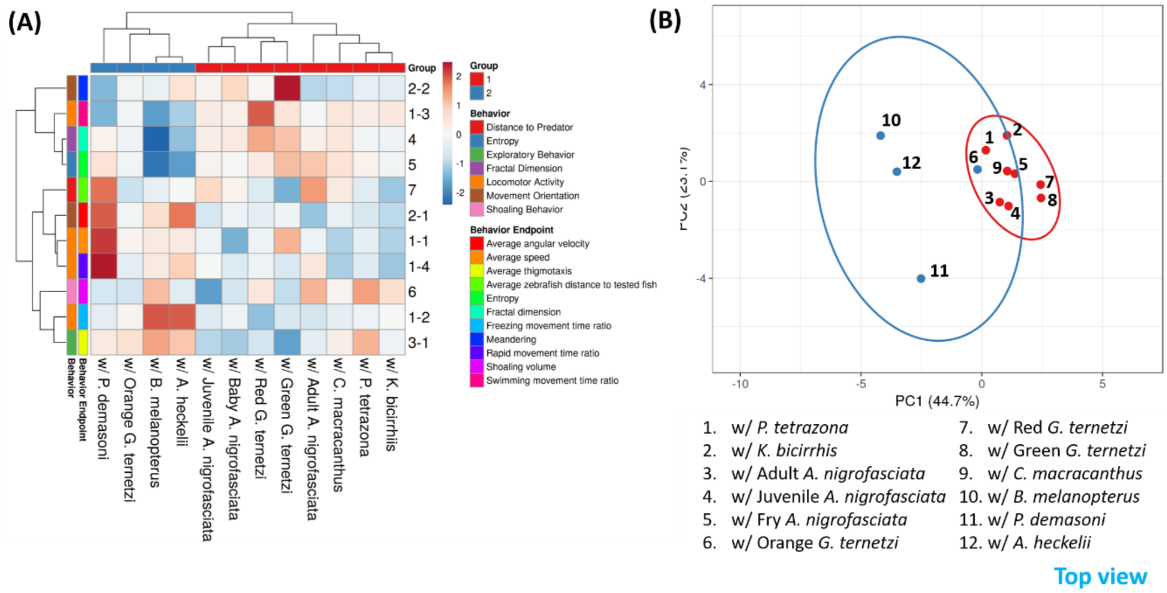


**Fig. S12. (A) Heatmap clustering analysis and (B) Principal component analysis (PCA) of all behavioral endpoints in zebrafish top view data after introduction to tested fishes.** Two major clusters were formed from the heatmap clustering marked with red (Group 1) and blue (Group 2).

**Table S1.** Summary of zebrafish behavioral endpoints measured in the experiment.

| Endpoint group | Behavior Endpoints (Units) | Definition |
|---|---|---|
| Locomotor Activity Endpoints | Average speed (cm $s^{-1}$) | Total distance traveled by fish divided by total time duration |
| | Freezing time ratio (%) | Total percentage of time when fish’s speed is less than 1 cm $s^{-1}$ |
| | Swimming time movement ratio (%) | Total percentage of time when fish’s speed is between 1 and 10 cm $s^{-1}$ |
| | Rapid movement time ratio (%) | Total percentage of time when fish’s speed is more than 10 cm $s^{-1}$ |
| Movement Orientation Endpoints | Average angular velocity (°/s) | Average magnitude of zebrafish angular speed |
| | Meandering (°/m) | Movement without a fixed direction or path |
| Exploratory Behavior Endpoints | Time in top (%) | Total percentage of time spent by zebrafish in the top portion of the novel tank |
| | Time in middle (%) | Total percentage of time spent by zebrafish in the middle portion of the novel tank |
| | Time in bottom (%) | Total percentage of time spent by zebrafish in the bottom portion of the novel tank |
| | Thigmotaxis (cm) | Average distance to the center of the novel tank |
| | Total distance traveled in top (cm) | Total distance traveled in the top portion of the novel tank |
| | Number of entries to the top | Zebrafish total entry occurrence to the upper half of the novel tank |
| Fractal Dimension | Fractal Dimension | Complexity of fractal patterns as a ratio of the change in detail to the change proportionally |
| Entropy | Entropy | Predictability of zebrafish movement |
| Shoaling behavior | Shoaling volume | Zebrafish shoaling behavior volume |
| Average distance to tested fishes | Average distance to tested fishes | Average distance of zebrafish to tested fishes |

**Table S2.** Comparison of tested fishes average body area size (Mean ± SD).

| Species | Body area ($cm^2$) | Species | Body area ($cm^2$) |
|---|---|---|---|
| *P. tetrazona* | 4.72 ± 0.83 | Red *G. ternetzi* | 3.91 ± 0.66 |
| *K. bicirrhis* | 7.40 ± 0.37 | Green *G. ternetzi* | 5.75 ± 0.93 |
| Adult *A. nigrofasciata* | 22.14 ± 2.41 | *C. macracanthus* | 4.27 ± 0.57 |
| Juv *A. nigrofasciata* | 8.78 ± 0.96 | *B. melanopterus* | 10.99 ± 1.35 |
| Fry *A. nigrofasciata* | 4.17 ± 0.75 | *P. demasoni* | 6.68 ± 1.07 |
| Orange *G. ternetzi* | 5.15 ± 0.67 | *A. heckelii* | 17.32 ± 1.60 |
